# Glucose Uptake and Its Effect on Gene Expression in *Prochlorococcus*

**DOI:** 10.1371/journal.pone.0003416

**Published:** 2008-10-20

**Authors:** Guadalupe Gómez-Baena, Antonio López-Lozano, Jorge Gil-Martínez, José Manuel Lucena, Jesús Diez, Pedro Candau, Jose Manuel García-Fernández

**Affiliations:** 1 Departamento de Bioquímica y Biología Molecular, Edificio Severo Ochoa, Campus de Rabanales, Universidad de Córdoba, Córdoba, Spain; 2 Instituto de Bioquímica Vegetal y Fotosíntesis, Universidad de Sevilla–CSIC, Sevilla, Spain; University of Wisconsin-Milwaukee, United States of America

## Abstract

The marine cyanobacteria *Prochlorococcus* have been considered photoautotrophic microorganisms, although the utilization of exogenous sugars has never been specifically addressed in them. We studied glucose uptake in different high irradiance- and low irradiance-adapted *Prochlorococcus* strains, as well as the effect of glucose addition on the expression of several glucose-related genes. Glucose uptake was measured by adding radiolabelled glucose to *Prochlorococcus* cultures, followed by flow cytometry coupled with cell sorting in order to separate *Prochlorococcus* cells from bacterial contaminants. Sorted cells were recovered by filtration and their radioactivity measured. The expression, after glucose addition, of several genes (involved in glucose metabolism, and in nitrogen assimilation and its regulation) was determined in the low irradiance-adapted *Prochlorococcus* SS120 strain by semi-quantitative real time RT-PCR, using the *rnpB* gene as internal control. Our results demonstrate for the first time that the *Prochlorococcus* strains studied in this work take up glucose at significant rates even at concentrations close to those found in the oceans, and also exclude the possibility of this uptake being carried out by eventual bacterial contaminants, since only *Prochlorococcus* cells were used for radioactivity measurements. Besides, we show that the expression of a number of genes involved in glucose utilization (namely *zwf*, *gnd* and *dld*, encoding glucose-6-phosphate dehydrogenase, 6-phosphogluconate dehydrogenase and lactate dehydrogenase, respectively) is strongly increased upon glucose addition to cultures of the SS120 strain. This fact, taken together with the magnitude of the glucose uptake, clearly indicates the physiological importance of the phenomenon. Given the significant contribution of *Prochlorococcus* to the global primary production, these findings have strong implications for the understanding of the phytoplankton role in the carbon cycle in nature. Besides, the ability of assimilating carbon molecules could provide additional hints to comprehend the ecological success of *Prochlorococcus*.

## Introduction

*Prochlorococcus* [1] constitutes an abundant group of marine cyanobacteria, being currently considered as one of the main global primary producers, since it is a significant part of the marine phytoplankton (recent estimates gave the number of 10^27^ *Prochlorococcus* cells on Earth), which in turn contributes roughly half of the net primary production in the biosphere, given that ca. half that population enters every day the trophic chain in the oceans [2]. The scarcity of nutrients in their natural habitats and the very low light available at depth were probably the main challenges faced by these organisms to colonize and become a predominant player in intertropical oceans. Several mechanisms have been proposed to explain this success: namely the development of specific, optimized photosynthetic machinery extremely efficient in the capture and utilization of the available light energy [3]; and the fine tuning of their metabolic pathways to focus only on the available nutrients, while removing non-essential genes, thus preventing unnecessary expenses of energy, in a process of evolutive genome compaction involving both gain and loss of genes [4]–[8]. For instance, all studied *Prochlorococcus* strains are devoid of enzymes which are almost universally found in other cyanobacterial groups, such as nitrate reductase [9], possibly due to the high energetic cost of assimilating nitrate.

In addition to the features discussed above, *Prochlorococcus* has been considered for long as an obligate photoautotrophic organism, performing photosynthesis to obtain energy and synthesize organic matter. Zubkov and co-workers reported the first evidences of mixotrophy in *Prochlorococcus*, showing the uptake of organic nitrogen compounds [10]–[13]. Dimethylsulfoniopropionate uptake has also been demonstrated in *Prochlorococcus* [14]. However the utilization of molecules containing only carbon skeletons has never been studied thus far, to the best of our knowledge. This could be probably explained by the very low concentrations of organic nutrients present in the large oligotrophic regions where *Prochlorococcus* thrives [15]. The values of dissolved organic matter (and sugars in particular) determined in the oceans reflect a steady state and can be very low [16], [17] because these compounds are being used very quickly upon release, so that there is a tight coupling between their production and utilization [18]. Due to these reasons, the possibility of sugar utilization by *Prochlorococcus* has been largely overlooked, and very little information is available on this matter. Nevertheless, under specific circumstances, and given that the production of dissolved organic matter is heterogeneous in time and space [19], [20], *Prochlorococcus* could benefit from the assimilation of organic molecules, such as sugars, that could be utilized as sources for carbon skeletons and/or to obtain reducing power, thus complementing the energetic income of the cells. Cyanobacteria differ greatly in their abilities to metabolize sugars [21]. In nature, cyanobacteria live under light/dark cycles. During the light period, they obtain energy from sunlight to produce organic matter by photosynthesis; under darkness, glucose residues from glycogen are catabolized via the oxidative pentose phosphate pathway, the final steps of glycolysis, and an incomplete tricarboxylic acid cycle, producing NAD(P)H and biosynthetic intermediates [21], [22]. The possibility of glucose utilization by *Prochlorococcus* was already proposed in an early study [23], based on the expression decrease of the photosynthetic gene *psbA* after glucose addition to cultures subjected to darkness. Yet, glucose consumption has never been specifically addressed in *Prochlorococcus* cells.

In this paper, we present the results of two kind of studies focused on glucose uptake and its effect on gene expression. First, radioactively labelled glucose was added to samples of different *Prochlorococcus* strains to check whether this sugar was being taken up; then *Prochlorococcus* cells were separated from contaminants by using a cell sorter coupled to a flow cytometer. These cells were used to determine glucose uptake. Second, we carried out gene expression studies on *Prochlorococcus* cultures after glucose addition. Uptake analysis were performed on high irradiance- and low irradiance-adapted ecotypes of *Prochlorococcus*, while expression studies were done in the low irradiance-adapted SS120 strain, considered to be a representative of ecotypes inhabiting environments where low light energy is available and therefore glucose utilization could be most useful.

## Results

### Characterization of glucose uptake in *Prochlorococcus*

As described in Materials and Methods, radiolabelled glucose (1 µM final concentration) was added to culture samples of high irradiance- and low irradiance-adapted strains. Then *Prochlorococcus* cells were sorted out by flow cytometry, and used to determine glucose uptake by filtration and radioactivity determination in a scintillation counter. We used stringent criteria for cell sorting, in order to avoid any kind of contaminants, so that radioactivity measured in uptake experiments was derived from *Prochlorococcus* cells exclusively (Supporting Figure S1).

**Figure 1:**
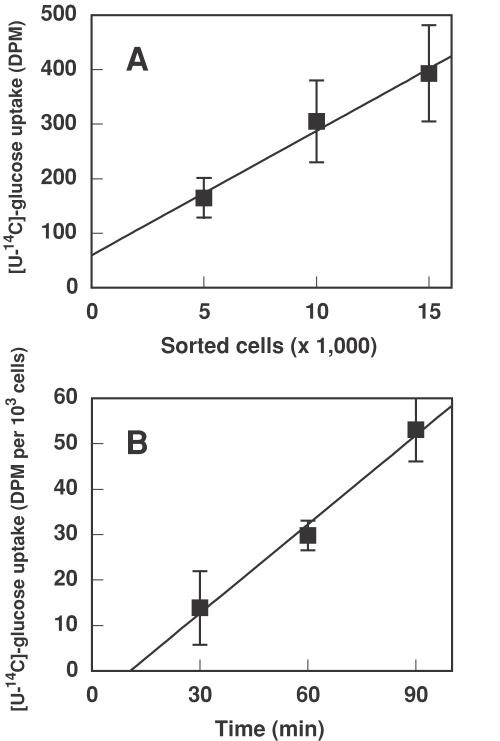
A. Glucose uptake *vs* number of *Prochlorococcus* PCC 9511 cells (axenic, high irradiance-adapted strain). Radiolabelled glucose, 1 µM final concentration, was added to *Prochlorococcus* cultures and incubated for 60 min in the light. Cells were sorted to obtain samples containing 5,000, 10,000 and 15,000 cells and glucose uptake was determined. B. Time course of glucose uptake in *Prochlorococcus* PCC 9511. Radiolabelled glucose, 1 µM final concentration, was added to *Prochlorococcus* cultures and samples taken at the indicated times and processed as described in Materials and Methods. Bars indicate the average from three independent experiments, each of them determined in triplicate, ±s.d.

**Figure 2:**
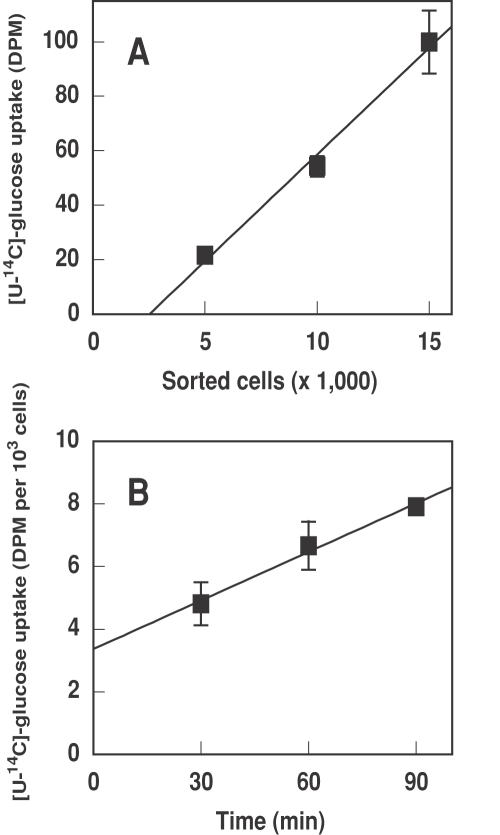
A. Glucose uptake *vs* number of *Prochlorococcus* SS120 cells (low irradiance-adapted strain). Radiolabelled glucose, 0.1 µM final concentration, was added to *Prochlorococcus* cultures and incubated for 60 min in the light. Cells were sorted to obtain samples containing 5,000, 10,000 and 15,000 cells and glucose uptake was determined. B. Time course of glucose uptake in *Prochlorococcus* SS120. Radiolabelled glucose, 0.1 µM final concentration, was added to *Prochlorococcus* cultures and samples taken at the indicated times and processed as described in Materials and Methods. Bars indicate the average from three independent experiments, each of them determined in triplicate, ±s.d.

**Figure 3:**
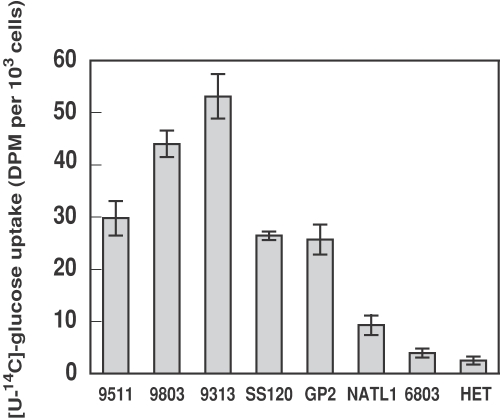
Glucose uptake by several *Prochlorococcus* strains: PCC 9511, TAK9803-2, MIT9313, SS120, GP2, NATL1-A, by *Synechocystis* sp. strain PCC 6803, and by heterotrophic bacteria sorted from a *Prochlorococcus* SS120 culture. Data are the average of the radiolabelled glucose (1 µM final concentration) taken up after 60 min incubation in the light, from three independent experiments, each of them determined in triplicate, ±s.d. PCC 9511 and TAK9803-2 are high irradiance-adapted strains; MIT9313, SS120, GP2 and NATL1-A are low irradiance-adapted strains.

**Figure 4:**
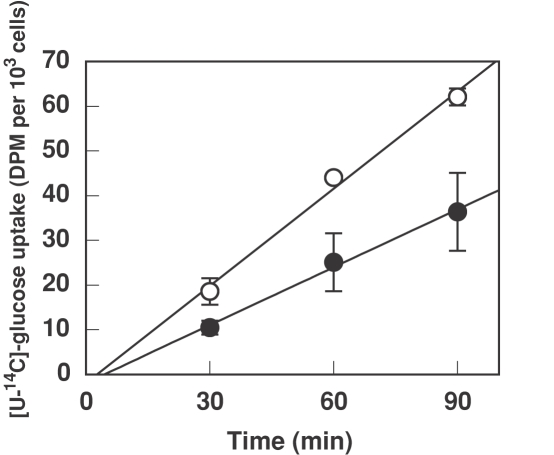
Time course of glucose uptake in *Prochlorococcus* SS120 samples under light (open circles) or darkness (closed circles). A *Prochlorococcus* culture was divided in two parts; one was ketp under the same light conditions, while the other was subjected to darkness for the rest of the experiment. After 24 h under such conditions, 1 µM radiolabelled glucose (final concentration) was added to both aliquots and processed as described in Materials and Methods. Samples were taken at the shown times. Bars indicate the average of three independent experiments, each of them determined in triplicate ±s.d.

The amount of glucose taken up by *Prochlorococcus* cells was strictly proportional to the number of cells sorted, as shown in Figure 1A, validating the method and discarding that any radioactivity other than that carried by *Prochlorococcus* cells was measured. Negative control experiments were carried out by using axenic *Prochlorococcus* PCC 9511 cells boiled for 5 min, in order to ensure that the determined uptake was the result of biological activity. The value from these controls (2.54±0.31 DPM per 10^3^ cells) has been substracted from the values corresponding to Figures 1, 2, 3 and 4 and Supporting Figure S2. Besides, negative control experiments were performed using *Nostoc* sp. strain PCC 7120 cells (an organism known to lack the capability for glucose uptake [24]), where the obtained values were very close to those corresponding to boiled *Prochlorococcus* cells (not shown).

Glucose uptake was linear for at least 90 min, as shown for *Prochlorococcus* PCC 9511 in Figure 1B; it must be noted that the strain PCC 9511 is axenic, being the first pure strain of *Prochlorococcus* obtained in the laboratory [25]. The same kind of experiments was carried out for all studied strains (see Supporting Figure S2). These results demonstrate that *Prochlorococcus* cells are actively taking up glucose, and that this uptake is not due to the activity of contaminants.

A lower glucose concentration (0.1 µM), closer to that observed in the oceans [15], [18] was also tested (Figure 2), finding that the uptake was also significant (ca. 5 fold lower than the uptake rates observed on 1 µM glucose). Therefore, our results indicate that glucose uptake can be efficiently carried out by *Prochlorococcus*, even at concentrations close to those observed in nature.

### Glucose uptake by different *Prochlorococcus* strains

Figure 3 shows the average uptake of radioactive glucose by several *Prochlorococcus* strains after 60 min incubation. Specific results for each strain are provided in Supporting Figure S2. All tested strains took up glucose at a rather high rate, although there was a 2 to 4-fold variation among them. We found no clear correlation between the rate of glucose uptake and the depth from which the tested *Prochlorococcus* strain was isolated. However, the large variations found and the low values of standard deviation within each strain, suggest that this could be a feature specific for the different *Prochlorococcus* isolates. As a reference, we have shown in Figure 3 the glucose uptake values obtained after FACS sorting of the heterotrophic population from a *Prochlorococcus* SS120 culture. It is worth noting that the values of glucose uptake are given on a per cell basis, and therefore the total amount of glucose taken up by the heterotrophs is much lower than that of *Prochlorococcus* since the number of contaminants is only a few percent of the total cells of the culture.

For a comparison, the uptake rate of the freshwater strain *Synechocystis* PCC 6803, capable to grow photoheterotrophically on glucose [26], [27], was also included, and found to be 2 to 10 times lower than those of *Prochlorococcus* cells. As stated above, it is worth noting that our comparative data are expressed in a per cell basis; even so, *Prochlorococcus* is taking up at least double amount of glucose than *Synechocystis* cells, which are much bigger. Should the data be expressed in concentration units, the differences would be even higher. Interestingly, it has been reported that the rate of amino acids uptake by *Prochlorococcus* is also 10-fold higher than by marine *Synechococcus* strains [12].

### Effect of darkness on glucose uptake in *Prochlorococcus* SS120

Our initial hypothesis considered that glucose uptake could be most useful for *Prochlorococcus* under conditions of energy limitation. To this purpose, we compared the rate of radiolabelled glucose incorporation in cultures of the low-irradiance adapted *Prochlorococcus* SS120 subjected to 24 h of darkness *vs* a control culture kept under standard light; the results are shown in Figure 4. The incubation of cells for 24 h in the dark intended to deplete their energy reserves, which would (according to our hypothesis) induce an increase in glucose uptake. However, and much to our surprise, the glucose uptake rate was found to be higher under light, with a ca. 40% decrease when cells were subjected to darkness. In good agreement with these results, light also enhances the uptake of sugars in other cyanobacteria [28], [29], and that of amino acids in *Prochlorococcus* (50% increase) [13], [29].

### Changes in gene expression after glucose addition to *Prochlororococcus* SS120 cultures

**Figure 5:**
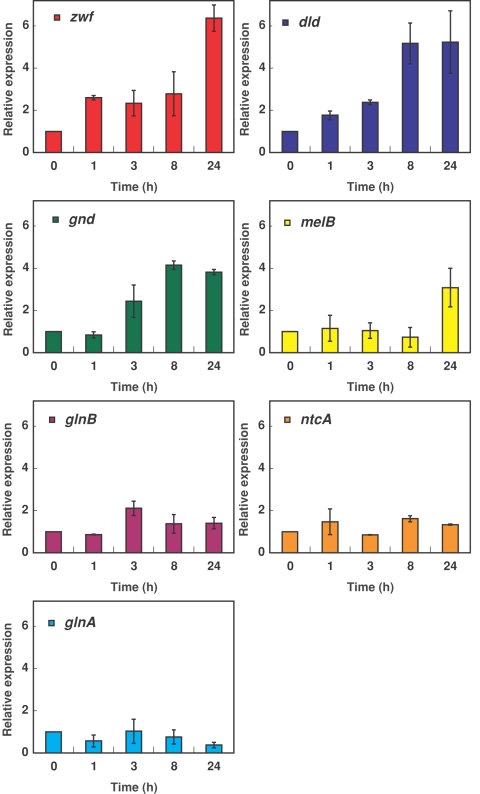
Effect of glucose addition on the gene expression of *Prochlorococcus* SS120, as monitorized by real time RT-PCR semi-quantitative analysis. Glucose (1 µM final concentration) was added to *Prochlorococcus* cultures, and samples were taken at the indicated times. Mathematical treatment as described in Materials and Methods. Expression level = 1 indicates no changes with respect to the control (no addition). Bars indicate the average of three independent determinations ±s.d.

Since the above reported results showed that *Prochlorococcus* was actively taking up glucose, we studied the effect of glucose addition on the expression of *Prochlorococcus* SS120 genes involved in different metabolic pathways, to find out if the addition of glucose induced changes in gene expression, and if so, to address whether it was a general transcriptional effect or specific for some of the studied genes (Figure 5). As described in Materials and Methods, we used the available genome of *Prochlorococcus* SS120 to design the primers for qRT-PCR quantification of gene expression. Furthermore, we performed, at the end of every experiment of gene quantification, a determination of the melting point of the amplified fragments, to ensure that only a single fragment of DNA is amplified in each tube. Besides, samples were subjected, after amplification, to electrophoresis to confirm the presence of a single band of the expected size in each tube. This strongly suggests that our amplifications are specific for *Prochlorococcus*.

For the sake of clarity, we only show the results obtained for seven of them: *glnA*, encoding glutamine synthetase; *ntcA* and *glnB*, encoding the regulatory proteins NtcA and P_II_ respectively (which control the expression of genes involved in the assimilation of nitrogen, in a process depending on the C/N balance [30]–[33]); *melB*, encoding a putative sugar transporter; *zwf* and *gnd*, encoding the enzymes glucose-6-phosphate dehydrogenase and 6-phosphogluconate dehydrogenase, participating in the pentoses phosphate pathway, which is the major route for catabolism of sugars in cyanobacteria [34]; and finally *dld*, encoding D-lactate dehydrogenase, which could participate in the regeneration of NAD^+^ from NADH, allowing the partial utilization of glucose [21], [35].

All genes showed some oscillations in the first hours, but we observed that glucose addition provoked significant increases in the expression of *zwf*, *dld* and *gnd* (ca. 6, 5 and 4-fold, respectively after 24 h of glucose addition), all of them involved in pathways of glucose metabolization. Interestingly, the expression of *zwf* was also increased early in studies on *Synechocystis* PCC 6803 during glucose feeding [36]. Besides, the expression of *melB*, a putative sugar transporter, was also increased 3-fold. Although the actual function of this gene annotated as *melB* in the *Prochlorococcus* genomes is not known, the clear increase in its expression after glucose addition to cultures suggests that it might be in fact involved in sugar uptake. By contrast, the expression of other genes not involved in glucose utilization (*glnA*, *ntcA*, Fig. 5; *glsF*, *icd*, not shown), had minor oscillations. Interestingly, the *glnB* gene experimented a certain increase (more than 2-fold after 3 h since glucose addition). This is consistent with the role of P_II_ (encoded by *glnB*) in the coordination of the carbon and nitrogen metabolisms in cyanobacteria [30].

Our studies on gene expression show that glucose provokes indeed an upregulation of genes directly involved in its utilization, a logic event from a physiological point of view, if glucose is to be utilized by *Prochlorococcus*. We are currently undertaking comparative proteomic studies to analyze the effects of glucose on *Prochlorococcus*, and preliminary observations seem to confirm that its addition induces changes in the proteome.

### Pathways for glucose utilization in *Prochlorococcus*

**Figure 6:**
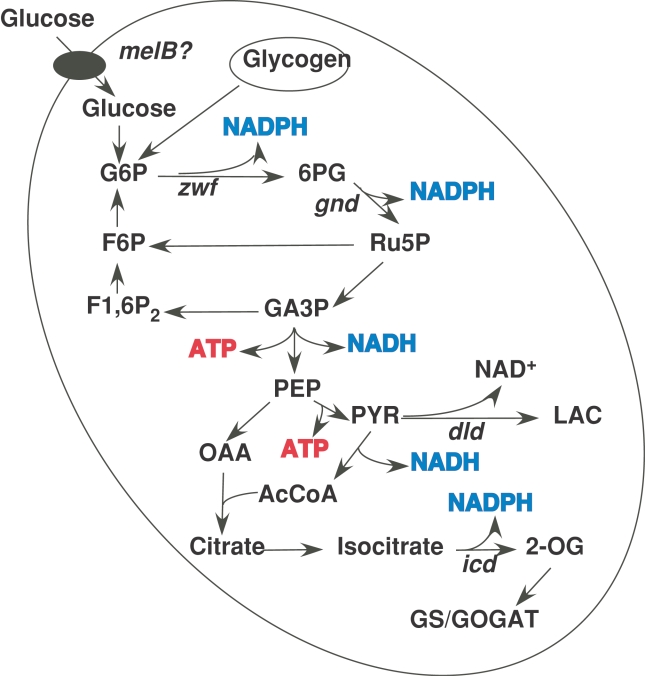
Pathways for glucose utilization repreented in the genome of *Prochlorococcus* SS120. The genes whose expression was quantified in this study are shown. The complete list of genes involved in these pathways and the corresponding enzymes are shown in Table 1. Abbreviations: G6P, glucose 6-phosphate; 6PG, 6-phosphogluconate; F6P, fructose 6-phosphate; Ru5P, ribulose 5-phosphate; F1,6P_2_, fructose 1,6-bisphosphate; GA3P, glyceraldehyde 3-phosphate; PEP, phosphoenolpyruvate; OAA, oxalacetate; PYR, pyruvate; LAC, lactate; AcCoA, acetyl-coenzyme A; 2-OG, 2-oxoglutarate.

Cyanobacteria are natural photoautotrophs. However, about half of the tested strains can also consume glucose or other carbohydrates for heterotrophic growth [27], [34]. Figure 6 outlines the metabolic pathways in *Prochlorococcus* SS120, enabling the utilization of glucose. All the genes encoding the enzymes catalyzing those reactions are present in the genome of *Prochlorococcus* SS120. In five of the enzymatic reactions, there is production of reducing equivalents (either NADPH or NADH); besides, net ATP is produced also as a result of the metabolization of glyceraldehyde-3-phosphate and phosphoenolpyruvate. This means that, even in the absence of a complete Krebs cycle (a feature shared by all cyanobacteria [37]), *Prochlorococcus* can actually obtain energy and reducing power from glucose. If we take into account that *Prochlorococcus* populations can live at depths down to 200 m, where there is very little energy input from sunlight, the utilization of glucose could be an advantage to survive under such conditions.

Table 1 shows a list of the proteins involved in the pathways shown in Figure 6; the corresponding genes are present in all *Prochlorococcus* genomes available thus far (with the exception of the gene *dld*, which is present only in the SS120 and MIT9211 strains), indicating that they possibly belong to the common core shared by the genus *Prochlorococcus* [8]. This, in combination with our results, strongly suggests that exogenous glucose can be utilized by the whole population of *Prochlorococcus*.

**Table 1:** Proteins involved in the pathways of glucose utilization outlined in Figure 6.

Protein	Gene	EC code	Gene code in SS120
Hypothetical sugar transporter	*melB*	-	Pro1404
Phosphoglucomutase	*pgm*	5.4.2.2	Pro0090
Glucokinase	*glk*	2.7.1.2	Pro1065
Glucose-6-phosphate isomerase	*pgi*	5.3.1.9	Pro0946
Glucose-6-phosphate dehydrogenase	*zwf*	1.1.1.49	Pro1124
6-phosphogluconolactonase	*nagB*	3.1.1.31	Pro0844
6-phosphogluconate dehydrogenase	*gnd*	1.1.1.44	Pro0843
Phosphoribulokinase	*prk*	2.7.1.19	Pro0861
Pentose-5-phosphate-3-epimerase	*rpe*	5.1.3.1	Pro0839
Ribose-5-phosphate isomerase	*rpi*	5.3.1.6	Pro1644
Transketolase	*tktA*	2.2.1.1	Pro1770
Transaldolase	*tal*	2.2.1.2	Pro0519
Triosephosphate isomerase	*tpi*	5.3.1.1	Pro0901
Glyceraldehyde-3-phosphate dehydrogenase	*gap3*	1.2.1.12	Pro1577
3-phosphoglycerate kinase	*pgk*	2.7.2.3	Pro0221
Phosphoglycerate mutase	*gpmB*	5.4.2.1	Pro0515
Enolase	*eno*	4.2.1.11	Pro0235
Piruvate kinase	*pykF*	2.7.1.40	Pro0923
Fructose-1,6-bisphosphatase aldolase I	*fda*	4.1.2.13	Pro0856
Fructose-1,6-bisphosphatase aldolase II	*cbba*	4.1.2.13	Pro0855
Fructose-1,6-bisphosphatase / Sedoheptulose 1,7-biphosphate phosphatase	*glpX*	3.1.3.11	Pro0840
Glucose-6-phosphate isomerase	*pgi*	5.3.1.9	Pro0946
Pyruvate dehydrogenase E1 alpha subunit	*acoA*	1.2.4.1	Pro1362
Pyruvate dehydrogenase E1 beta subunit	*acoB*	1.2.4.1	Pro0766
Pyruvate dehydrogenase E2 component (Dihydrolipoamide S-acetyltransferase)	*aceF*	2.3.1.12	Pro0401
Dihydrolipoamide dehydrogenase	*lpd*	1.8.1.4	Pro1372
Citrate synthase	*gltA*	2.3.3.1	Pro0185
Aconitase	*acnB*	4.2.1.3	Pro1866
Isocitrate dehydrogenase	*icd*	1.1.1.42	Pro1752
Phosphoenolpyruvate carboxylase	*ppc*	4.1.1.31	Pro1730

Gene codes are shown for the *Prochlorococcus* SS120 genome, but all of them are present in the available *Prochloroccus* genomes.

## Discussion

The results presented in this paper evidence that *Prochlorococcus* is capable of glucose uptake and strongly suggest its utilization, in contrast to its previously accepted status as a strictly autotrophic organism. Mixotrophy is known long ago in cyanobacteria [27], and both glucose [34] and amino acid [38] utilization have been reported in different cyanobacterial strains. The uptake of amino acids has been previously described in *Prochlorococcus* [10]–[13], [29], [39], providing initial evidence for mixotrophy in these cyanobacteria, but nitrogen and not carbon was considered to be the main interest of that uptake, since nitrogen is often a limiting nutrient in oligotrophic environments. The discovery of organic carbon molecules being also taken up by *Prochlorococcus* has deep consequences in ecological terms. The *Prochlorococcus* consideration with regard to the global carbon cycle should take into account that, besides being a primary producer when performing photosynthesis, it could also utilize organic carbon molecules. This shows different behaviours depending on the environmental conditions. In addition, it must be stressed that no contribution to fulfill the standard nutrient limitations in the oceans (N, P, Fe) can be drawn from glucose, thus suggesting that the benefit could derive from the reducing power and ATP obtained by the metabolization of glucose (Fig. 6).

In order to discard the possibility that our results could be derived from *Prochlorococcus* incorporation of radiactive CO_2_, previously released to the media after glucose was used for respiration by heterotrophic bacteria, we carried out some calculations: since our *Prochlorococcus* samples contained ca. 1.1×10^7^ cells/ml, with an average uptake rate of 0.05 fmol/cell, they would take up 0.55 nmol glucose in 1 hour. Since we added 1 nmol of glucose to samples, it means that *Prochlorococcus* cells were taking roughly 55% of the added gluose in 1 h. Considering that we have, at most, 3% of contaminant heterotrophic bacteria in non axenic *Prochlorococcus* samples, it means that, to account for the hypothesis here analyzed, they should excrete 33-fold more radiolabelled C per cell than it is taken up by *Prochlorococcus*. In turn, this means they should have an uptake ratio even higher (given they would not excrete all the radiolabelled carbon coming from glucose). Furthermore, even accepting the idea that bacteria excreted all the carbon from glucose, each heterotrophic bacterium should excrete 1.2×10^−13^ g of C per hour to produce the values observed by us, which represents an amount of C 8–10 times higher than the total C content of such cell (i.e., approx 10^−14^ g of C).

On the other hand, if we assumed an uptake rate for heterotrophic bacteria 10 times higher than that for *Prochlorococcus*, it would lead, in the first measured sample at 30 min, to an uptake of 85 pmol of glucose, and if all that glucose was excreted, it would give a concentration of 85 nM. However, we measure an uptake value of 275 pmol. Consequently, these simple calculations evidence that our results can not be explained on the base of an hypothetical uptake of CO_2_ excreted by heterotrophic bacteria and later incorporated into organic matter in *Prochlorococcus* by means of photosynthesis.

It is worth noting that glucose uptake is linear for, at least, 90 min (Figs. 1 and 4, Supporting Figure S2), indicating its metabolization, since otherwise the internal glucose concentration after 60 min would build up to very high level, in the order of 0.1 M or higher, which is far above the K_m_ values from known enzymes utilizing glucose represented in cyanobacteria (i.e., 0.1–0.5 mM in the case of glucokinases) and could even pose an osmotic problem.

Glucose can not be oxidized in *Prochlorococcus* through the Krebs cycle, since cyanobacteria have been known for long to posses an incomplete cycle lacking 2-oxoglutarate dehydrogenase [37]. In addition, *Prochlorococcus* genomes lack the gene encoding the 6-phosphofructokinase, which is present in other cyanobacterial genomes, thus preventing the transformation of fructose-6-phosphate into fructose-1,6-bis-phosphate, an essential step of the glycolytic pathway. This gene is also absent in marine *Synechococcus*, *Gloeobacter violaceus*, *Trichodesmium erythraeum* (as deduced from their genomes) and *Synechocystis* PCC 6308 [34]. However, a complete oxidative pentose phosphate pathway is present in all *Prochlorococcus* genomes, meaning that glucose could be fully oxidized. Glyceraldehyde-3-phosphate and fructose-6-phosphate could be produced from pentoses phosphate through the action of transaldolase and transketolase, giving in turn glucose-6-phosphate that could undertake another decarboxylation cycle. In this way, twelve molecules of NADPH could be produced per glucose (Fig 6). The production of reducing power could be important in *Prochlorococcus*, under conditions of strong energetic limitations. In the case of strains possessing the *dld* gene (as SS120), encoding D-lactate dehydrogenase, it could be used to obtain 2 molecules of ATP per glucose metabolized, by regeneration of NAD^+^, as we have previously proposed [35]. Additional work is necessary to confirm this hypothesis. It is generally accepted that in vivo NAD-dependent lactate dehydrogenase from cyanobacteria functions in the conversion of pyruvate to lactate. Excretion of D-lactate under dark anoxic conditions as an end product of endogenous carbohydrate catabolism has been reported for *Synechococcus* PCC 6716 [21], [40].

Furthermore, this could provide an explanation for the maintenance of *Prochlorococcus* populations in seasons when they almost disappear at some specific environments. The utilization of organic carbon could allow to survive at very low metabolic rates, by providing a small but appreciable source of energy and reducing power, even under conditions that make impossible to survive from photosynthesis alone (i.e., at depth in periods of very low surface irradiance). In this way, a tiny population would persist until the conditions become more favourable, enabling it to colonize again the same environment, as reported [41]. The glucose uptake in high irradiance-adapted ecotypes would be less relevant, since these cells are living in an environment that is not limited in irradiance. Future studies will address the actual utility of glucose uptake by *Prochlocoroccus*, since we have not yet studied this topic experimentally.

With regard to the big picture of the metabolic adaptations, the results here reported indicate that the genes required for exogenous glucose uptake and metabolization belong to the core set of conserved genes, seemingly occurring in all *Prochlorococcus* ecotypes [8]. In good agreement with this idea, the gene *melB* is present in all *Prochlorococcus* genomes thus far available. This fact strongly suggests that the importance of sugar uptake is high for all *Prochlorococccus* strains. Furthermore, the adaptive response sensory histidine kinase 8 (EC 2.7.13.3), required for heterotrophic growth in *Synechocystis* PCC 6803 [22], is present also in all available *Prochlorococcus* genomes. Interestingly, the gene *dld* appears only in the low irradiance-adapted strains SS120 and MIT9211, suggesting it could be useful under conditions of energy and oxygen limitation [35]. It is worth noting that *Prochlorococcus* is the dominant organism in the deep clorophyl maximum found in oxygen minimum zones [42], [43].

Given that metabolic pathways have been extensively modified along the evolution of *Prochlorococcus*, the widespread glucose utilization in this microorganism suggests that this process is very important for its survival. Besides, it is tempting to speculate that, contrary to expectations, the utilization of glucose by *Prochlorococcus* -even in the very oligotrophic environments, which are its natural habitat- is worthwhile. Although it is known that glucose is often the only free neutral sugar detectable in the oceans [44], there is little information regarding its actual concentration [45]. In addition, its rapid uptake by the heterotrophic and mixotrophic organisms inhabiting those environments could provoke a strong underestimation of its availability.

A potentially important outcome of this study is the possibility of some degree of mutualism between *Prochlorococcus* and coexistent bacteria in nature; although the presence of sugars is not a condition for *Prochlorococcus* to grow (as evidenced by the utilization of artificial media without any sugar to culture *Prochlorococcus* [46]), it might contribute to a better development of cells, thus explaining the often difficult and unpredictable growth of this cyanobacterium in the laboratory. It is noteworthy that glucose addition to the first axenic strain of *Prochlorococcus*, PCC 9511, was found to be tolerated and seemed to slightly prolong survival [25].

The results reported here point out to an unexpected behaviour in *Prochlorococcus*. This is the first study, to our knowledge, providing evidence for sugar uptake and strongly suggesting its utilization in *Prochlorococcus*. While the actual utility of this glucose uptake remains to be elucidated, we can conclude that the current consideration of *Prochlorococcus* as one of the paramount primary producers on a global scale should be complemented. Rather than constraining its ecological contribution to that role, we should consider *Prochlorococcus* as evolved cyanobacteria whose genomes have been subjected to a large specialization [5], [7]. This enables them to remain competitive at the specific environment inhabited by each ecotype, by removing unnecessary genes, and keeping only the essential ones for survival, including the utilization of different nutrients (both organic, as sugars, and inorganic) in different environments. In this model, *Prochlorococcus* appears as a mixotrophic organism, relying mainly on autotrophy, but willing and able to take up organic compounds (i. e., amino acids and sugars) from the environment when available. While mixotrophy in oceanic protists [47] and bacteria [48] is well established, it has not been considered as a standard feature in cyanobacterial picophytoplankton thus far [47]. Although glucose uptake has not been tested in marine *Synechococcus* strains, the presence of putative sugar transporters (as *melB*) in their genomes, and the overall metabolic similarities between *Synechococcus* and *Prochlorococcus*, all suggest this might be the case. Therefore, we propose that mixotrophy is a widespread feature in oceanic picophytoplankton, in good agreement with recent reviews supporting this hypothesis [48]. The ecological relevance of this proposition remains open for study in the future, but it seems plausible to draw important consequences on the global carbon flow models in the ocean.

## Materials and Methods

### Strains and culturing

*Prochlorococcus marinus* strains PCC 9511, TAK9803-2 (high irradiance-adapted) and GP2, SS120, MIT9313 and NATL1A (low irradiance-adapted) were routinely cultured in polycarbonate *Nalgene* flasks using PCR-S11 medium as described by Rippka and coworkers [25]. The seawater used as the basis for this medium was kindly provided by the Instituto Español de Oceanografía (Spain). Cells were grown in a culture room set at 24°C under continuous blue irradiance (40 or 4 µmol quanta m^−2^ s^−1^ for high- and low-irradiance adapted strains, respectively). Culture growth was determined by measuring its absorbance at 674 nm. *Synechocystis* sp. strain PCC 6803 was cultured as described elsewhere [49], in BG-11 medium. For experiments requiring darkness, culture bottles were completely wrapped with two layers of aluminium foil, and the sampling was performed in the dark. For experiments addressing the changes in gene expression induced by glucose addition, 1 µM glucose (final concentration) was added to one of them, while another aliquot from the same original culture with no addition was used as control, and samples were taken from both carboys at the indicated times.

### Flow cytometry analysis of cell samples

*Prochlorococcus* was enumerated in freshly unstained samples by using its specific chlorophyll autofluorescence. One millilitre samples from each *Prochlorococcus* culture were used for analysis. When required, cells were fixed with 10% paraformaldehyde [50]. Cell counting was performed following the method described by Marie and coworkers [51], by using an *Epics XL* flow cytometer equipped with the software *Expo 32 ADC* and *Expo 32 Analysis*, from *Beckman Coulter*. The cytometer was calibrated by using 1 µm diameter fluorescent beads (*Flow Check High Intensity Green Alignment*, *Polysciences Inc*). From each sample 10,000 events were analyzed. As internal reference, 1 µL of a freshly prepared dilution of the beads solution (1 drop in 5 mL of filtered distilled water) was added to each culture sample. Forward scatter *vs* red fluorescence (i.e., emission between 660–700 nm, corresponding to the pigment composition of *Prochlorococcus* cells) was used to draw charts to identify the *Prochlorococcus* populations and the possible contaminants.

### Fluorescence-activated cell sorting (FACS)

*Prochlorococcus* cells were flow sorted utilizing a *FACS Vantage* flow cytometer (*Becton Dickinson*) equipped with *CellQuest Pro* software. The reproducibility of gate sorting was checked in triplicate, using three samples from different cultures. Time course glucose uptakes were measured by sorting 15×10^3^ *Prochlorococcus* cells at several times. Sorted cells were collected onto 0.22 µm filters (*Millipore*), washed with 25 mM Tricine-KOH pH 8.1 buffer and radioassayed. Typical settings were as follows: SSC = 400; FL3 = 700; all parameters were set on logarithmic amplification. Discriminator was set on FSC with a threshold of 100. Nozzle diameter was 70 µm. The working flow was adjusted to reach ca. 1,000 events/s in order to avoid coincidences while counting [51], [52]. Gating was done on FL3 acquisition. Enumeration of heterotrophic bacteria was carried out by staining samples with *SYBR-Green I* in a 1∶10,000 dilution [53].

### Glucose uptake determination

Cultures were kept at 24°C under 4 µmol quanta m^−2^ s^−1^ blue light as described [54]. [U-^14^C]-glucose (281 mCi/mmol, *Sigma*) was added to 1 mL of culture sample to reach a final concentration of 0.1 µM or 1 µM, depending on the experiment, and aliquots were taken at the indicated times, sorted and filtered through 0.22 µm *Millipore* filters. Once filtered, the samples were washed with 25 mM Tricine-KOH pH 8.1 and then introduced into scintillation counting vials. Scintillation was started by the addition of 3 mL *Ready Protein Cocktail* (*Beckman Coulter*) in a *LS6000IC Scintillation Counter* (*Beckman Coulter*). Under our experimental conditions, one pmol of radiolabelled glucose corresponds to 623.82 disintegrations per minute (DPM).

### RNA isolation

Cells were harvested by centrifugation at 30,100×*g* for 5 min at 4°C. After pouring most of the supernatant and carefully pipetting out the remaining medium, the pellet was directly resuspended in cold buffer (10 mM sodium acetate pH 4.5, 200 mM sucrose, 5 mM EDTA). Cells obtained from 500 mL of culture were resuspended in 250 µL of this buffer and immediately frozen and stored at −80°C until used. For RNA isolation the kit *Aurum Total RNA Mini Kit* from *Bio-Rad* was used, following the instructions from the manufacturer.

### Semi-quantitative real time RT-PCR determination of gene expression changes induced by glucose addition

Reverse transcription of RNA samples was carried out using the *iScript cDNA Synthesis Kit* from *Bio-Rad* following the instructions of the manufacturer. Real time amplification was monitorized by addying SYBR Green I/fluorescein (*Bio-Rad*) to samples, using an *iCycler iQ Multi-Color Real Time PCR Detection System* equipped with the software *iCycler iQ* v 3.0, from *Bio-Rad*.

PCR amplification samples (25 µL) contained 0.2 mM dNTPs, 0.6 µL of a *SYBR Green I*/fluorescein 10^−4^ dilution in DMSO, 0.4 units of *Taq* polymerase, 2.5 µL PCR buffer 10× (both provided by *BioTools*, Madrid, Spain), and different primers concentrations for each gene (depending on their efficiencies, which were previously calculated in order to optimize the amplification reactions [55]). Primers were designed utilizing the *Oligo v. 4.05 software* (*Molecular Biology Insights*), on the basis of the *Prochlorococcus* SS120 genome (whose sequences were retrieved from the *CYORF* database, available at http://cyano.genome.ad.jp/). The primers are described in Table 2.

**Table 2:** List of primers utilized in quantification of gene expression by semi-quantitative RT-PCR.

Gene	Protein	Primer	Sequence
***dld***	D-lactate dehydrogenase	Forward	TCGCTGTTTTTGCTGTAAG
		Reverse	TTAAATGCGTCAAATGTTC
***glnA***	Glutamine synthetase	Forward	GCGTCTTGTTCCTGGCTTC
		Reverse	AGCATCTCCTGACCTGAACTC
***glnB***	P_II_ protein	Forward	TTTGGGCGACAAAAAGGA
		Reverse	TCAACACTTTCATCAGCAACAA
***gnd***	6-phosphogluconate dehydrogenase	Forward	AAAGCAGGTCAAAAAGGAA
		Reverse	AGAAGCGTAAATGGTAGGG
***melB***	Hypothetical sugar transporter	Forward	GCTTTTATGGCAGGTTCTTT
		Reverse	CAAATAGCCGCAAGACTCAG
***ntcA***	NtcA	Forward	AGCTCCTGCTGGCTCAGTTA
		Reverse	GAGAAGTAGCCCAACCCCAC
***rnpB***	Ribonuclease P	Forward	CTCTCGGTTGAGGAAAGTC
		Reverse	CCTTGCCTGTGCTCTATG
***zwf***	Glucose-6-phosphate dehydrogenase	Forward	ACGAGAAGCGATGAGGTAG
		Reverse	ATAAGGATAAGTTGGAAGTTG

The amplification protocol was optimized to ensure that a single amplification product was obtained. This was monitorized by electrophoresis, observing that all primers pairs give rise to the appearance of a single band, so that all fluorescence detected by the optical system of the *iCycler* thermocycler was produced only by the specific product of amplification.

The thermal amplification protocol was as follows: 1 cycle at 95°C for 5 min, 35 cycles of: 95°C for 15 s, 58°C for 30 s and 72°C for 30 s. After this protocol was ended, a melting point calculation protocol was routinely done in order to check that only the correct, single product was amplified in each tube. The expression of *rnpB* was used as internal standard to normalize the values obtained for all other genes. The mathematical treatment of data to calculate relative gene expression was performed according to Pfaffl [55], so that the expression was calculated by using the formula:
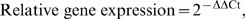
where ΔΔCt corresponds to the increase in the threshold cycle of the problem gene with respect to the increase in the threshold cycle of the housekeeping gene (*rnpB*, in this study). Hence, the final quantification value for each condition indicates the relative change of gene expression in the problem tube with respect to the control tube, for each sample. According to this formula, values of 1 mean no change; values >1 mean increase in gene expression; and values comprised between 0 and 1 mean decrease in gene expression.

### Sequence analysis

*Prochlorococcus* gene sequences were retrieved from the CYORF (http://cyano.genome.ad.jp/) and Integrated Microbial Genomes (http://imgweb.jgi-psf.org/cgi-bin/w/main.cgi) websites. Sequence similarities were analyzed by using BLAST [56] at the Microbes Online website (http://www.microbesonline.org/). Comparisons of the existing metabolic pathways in different *Prochlorococcus* strains were carried out using the Kyoto Encyclopedia of Genes and Genomes (KEGG, http://www.genome.ad.jp/kegg/pathway.html).

## Supporting Information

Figure S1 Typical side-scatter (SSC) vs red fluorescence plot of an unstained, live culture of *Prochlorococcus* MIT9313 (FACSVantage flow cytometer) utilized to draw the gating for cell sorting. R1 corresponds to the population of *Prochlorococcus* MIT9313; R2 corresponds to heterotrophic contaminant bacteria. Similar plots were used for the rest of strains utilized in this work.
(1.15 MB TIF)

Figure S2 A. Glucose uptake vs number of *Prochlorococcus* cells, in different strains. Radiolabelled glucose, 1 µM final concentration, was added to *Prochlorococcus* cultures and incubated for 60 min in the light. Cells were sorted to obtain samples containing 5,000, 10,000 and 15,000 cells and glucose uptake was determined. B. Time course of glucose uptake in *Prochlorococcus* strains. Radiolabelled glucose, 1 µM final concentration, was added to *Prochlorococcus* cultures and samples taken at the indicated times and processed as described in Materials and Methods. Bars indicate the average from three independent experiments, each of them determined in triplicate, ±s.d.
(0.08 MB EPS)
