# Supplementary figures and images for: Glucose Uptake and Its Effect on Gene Expression in Prochlorococcus
Source: PLoS One. 2008 Oct 20;3(10):e3416. doi: 10.1371/journal.pone.0003416 (PMC2565063; doi:10.1371/journal.pone.0003416)

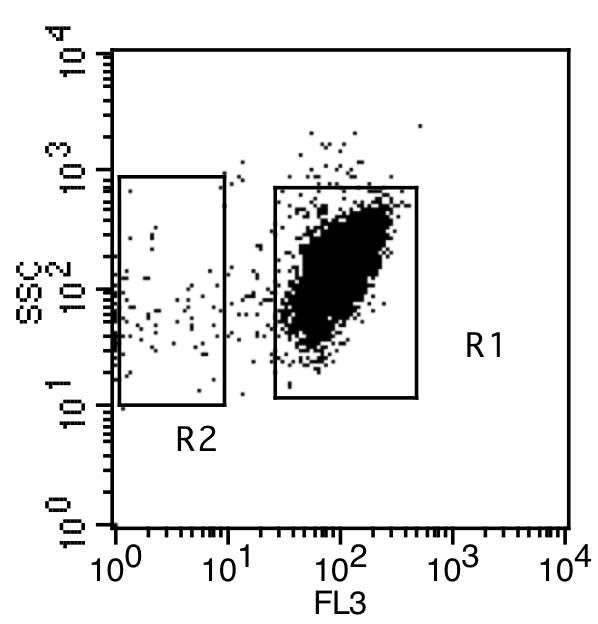

Supplement: Figure S1 — Typical side-scatter (SSC) vs red fluorescence plot of an unstained, live culture of Prochlorococcus MIT9313 (FACSVantage flow cytometer) utilized to draw the gating for cell sorting. R1 corresponds to the population of Prochlorococcus MIT9313; R2 corresponds to heterotrophic contaminant bacteria. Similar plots were used for the rest of strains utilized in this work. (1.15 MB TIF) [file pone.0003416.s001.tif]
